# Improving the prevention, diagnosis and treatment of TB among people living with HIV: the role of operational research

**DOI:** 10.1186/1758-2652-14-S1-S5

**Published:** 2011-07-06

**Authors:** Delphine Sculier, Haileyesus Getahun, Christian Lienhardt

**Affiliations:** 1 Stop TB Department, World Health Organization, 20 Avenue Appia, 1211 Geneva, Switzerland; 2 Stop TB Partnership, World Health Organization, 20 Avenue Appia, 1211 Geneva, Switzerland

## Abstract

Operational research is necessary to improve the access to and delivery of tuberculosis prevention, diagnosis and treatment interventions for people living with HIV. We conducted an extensive review of the literature and reports from recent expert consultations and research-related meetings organized by the World Health Organization and the Stop TB Partnership to identify a TB/HIV operational research agenda. We present critical operational research questions in a series of key areas: optimizing TB prevention by enhancing the uptake of isoniazid preventive therapy and the implementation of infection control measures; assessing the effectiveness of existing diagnostic tools and scaling up new technologies; improving service delivery models; and reducing risk factors for mortality among TB patients living with HIV. We discuss the potential impact that addressing the operational research questions may have on improving programmes’ performance, assessing new strategies or interventions for TB control, or informing global or national policy formulation. Financial resources to implement these operational research questions should be mobilized from existing and new funding mechanisms. National TB and HIV/AIDS programmes should develop their operational research agendas based on these questions, and conduct the research that they consider crucial for improving TB and HIV control in their settings in collaboration with research stakeholders.

## Background

From 1995 to 2004, the estimated incidence of tuberculosis (TB) increased steadily around the world, with an excess in sub-Saharan Africa, in relation with the emergence and expansion of the HIV epidemic [1]. The estimated TB incidence has been slowly declining since then, at a rate of 1% per year, making it impossible to reach the 2050 TB elimination target, defined as less than one TB case per million population [2]. There were an estimated 9.4 million incident TB cases in the world in 2009, of which an estimated 1.1 million (12%) were living with HIV; 1.7 million deaths were also estimated, including 380,000 among HIV individuals [3]. TB continues to be a leading cause of deaths among people living with HIV, accounting for one-fifth of the 1.8 million AIDS-related deaths in 2009.

While HIV testing among TB patients has rapidly scaled up since 2002, the uptake of antiretroviral therapy (ART) among TB patients living with HIV is unsatisfactory, with only 140,000 TB patients with HIV co-infection placed on ART worldwide in 2009 [3]. Prevention and treatment of TB among people living with HIV remains limited: only 5% of the estimated 33.3 million people living with HIV (about 1.7 million) were screened for TB in 2009, and less than 1% (88,000) of those found without active TB were enrolled on isoniazid preventive therapy (IPT) [3]. These percentages are still very far from the 100% and 50% targets set for 2015 in the Global Plan to Stop TB 2011-2015 [4]. To boost TB control activities in relation with HIV, and reach these targets, important operational research questions need to be urgently addressed.

The aim of this paper is to review and present the priority operational research questions to improve the prevention, diagnosis and treatment of TB among people living with HIV, including innovative models of service delivery for collaborative TB/HIV activities. Several definitions for operational research have been proposed [5-8]. For the purpose of this paper, we defined operational research according to its ultimate objectives, that are: (i) to improve programme performance and outcomes; (ii) to assess the feasibility, effectiveness or impact of new strategies or interventions for TB control; or (iii) to collect data to guide policy recommendations on specific interventions [9].

## Methods

We used a variety of sources of information. We reviewed PubMed and Web of Science databases using a combination of “tuberculosis” and “HIV” with the following key words: drug-resistant tuberculosis, anti-tuberculosis treatment, treatment of latent infection, antiretroviral therapy, infection control, laboratory services, childhood tuberculosis, pregnant women, community, programme evaluation, and operational research.

We also reviewed reports of expert consultations and other research-related meetings with a focus on operational research aspects organized by the World Health Organization (WHO) and the Stop TB Partnership in 2009-2010 [10-16]. The objectives and proceedings of these meetings included questions addressing the translation gaps in implementing existing and new tools and technologies within TB services, as well as questions on how operational research could best inform policy decision making at national, regional and global levels. The meetings and documents also defined key areas of TB control for which various research questions were to be addressed as priorities, taking into account the various elements of time, place and person, and issues of relevance and feasibility [16]. In one document, the TB/ HIV research questions were identified by various experts and prioritized using a scoring system that used a set criteria of effectiveness, deliverability, answerability and equity [17].

We have re-examined the documents for the aspects of operational research and present our findings in the following key areas: (i) prevention of TB among people living with HIV; (ii) implementation of infection control measures; (iii) early diagnosis and treatment of TB in people living with HIV; (iv) service delivery of collaborative TB/HIV activities; (v) reducing mortality among TB patients living with HIV; and (vi) childhood TB and HIV.

## Key areas of operational research

The WHO policy on collaborative TB/HIV activities recommends a combination of measures to reduce the burden of TB among people living with HIV [18]. These measures include intensified TB case finding, prevention of TB with IPT and ART, and efficient infection control, which are branded as the *Three 1’s for HIV/TB*. Their implementation requires sound policy and a programme environment that gives due consideration to the local context, the epidemiology of TB and HIV, and the status of health systems and infrastructure that will determine the service delivery models. In addition to the operational difficulties con fronted in providing quality and effective interventions, the cultural and system-wide differences between TB and HIV programmes, care providers and partner organizations may have contributed to the limited implementation of collaborative TB/HIV activities.

Operational research plays a key role in addressing these challenges and optimizing and scaling up prevention, diagnosis and treatment of TB among people living with HIV through effective service delivery models, including community-based interventions. Table 1 summarizes the operational research priorities to improve the implementation of collaborative TB/HIV programme activities [16,17,19]. The ultimate objective of each operational research question is presented, as well as how it influences the design and methodology of the research, the setting where it is conducted, the degree of generalizability of the results, and the ultimate relevance for TB control from local to global level [9].

**Table 1:** Priority operational research questions to optimize the implementation of collaborative TB/HIV activities

Question	Objective	Design/methodology	Setting	Generalizability	Relevance
**1. Prevention of TB among people living with HIV**					
In people living with HIV, what is the optimal TB screening algorithm to be used to safely initiate preventive TB therapy across settings with different burdens of TB and HIV disease?	To assess the feasibility, effectiveness and impact of the new WHO clinical algorithm for TB screening and scale up of IPT among people living with HIV, including children, pregnant women, those on ART and after completion of TB treatment	Interventional quantitative	HIV healthcare facilities and communities	Across settings with analytical approach needed to assess for heterogeneity	Regional, national
What are the best models for IPT delivery, clinical monitoring and community support to reduce drop out, incidence of breakthrough TB, and occurrence of severe adverse events?	To improve programme performance and outcomes; to assess feasibility and effectiveness of various models of adherence and monitoring	Observational qualitative; interventional quantitative	HIV healthcare facilities and communities	Setting specific; across settings with analytical approach needed to assess for heterogeneity	Regional, national, local
In people living with HIV, what is the optimal duration and timing of initiation of IPT in relation to ART to reduce the risk of active TB, compared with IPT or ART alone, in terms of safety, efficacy and cost effectiveness?	To collect data to guide policy recommendations on specific interventions	Interventional quantitative	TB and HIV healthcare facilities and communities	Multí settings/ countries using generic operational research protocol	Global, regional
Which health programmes (i.e.,TB, AIDS, mother and child, or other) can best lead the implementation of IPT for people living with HIV?	To improve programme performance and outcomes; to collect data to guide policy recommendations on specific interventions	Observational qualitative; interventional quantitative	TB, AIDS, mother and child, or other health programmes	Setting specific; multi settings/countries using generic operational research protocol	Global, local

**2. Implementation of infection control measures**					
What are the best infection control measures that effectively reduce TB transmission in healthcare and congregate settings, at home and in the community?	To improve programme performance and outcomes	interventional quantitative	TB and HIV healthcare facilities, congregate settings, households and communities	Setting specific	National, local
What are the best operational models (i.e., practical, feasible, easily reproducible and effective) to implement and monitor infection control measures in health facilities?	To improve programme performance and outcomes	interventional quantitative	TB and HIV healthcare facilities	Setting specific	National, local

**3. Early diagnosis and treatment of TB among people living with HIV**					
What is the feasibility, effectiveness and impact of currently available and new tools for rapid TB diagnosis and treatment among people living with HIV (including diagnosis of drug resistance and smear-negative disease)?	To assess feasibility, effectiveness and impact of existing and new TB tools and technologies; to collect data to guide policy recommendations on specific interventions	Interventional quantitative	TB and HIV healthcare facilities up to decentralized level	Multi settings/ countries using generic operational research protocol	Global, regional, national
What are the best combinations of diagnostic tools to enhance TB case finding among people living with HIV in HIV service facilities and at community level, in both high and low HIV prevalence settings?	To improve programme performance and outcomes	interventional quantitative	TB and HIV healthcare facilities, and communities	Setting specific	National, local
What are the best models to scale up the early and combined initiation of ART in TB patients living with HIV to reduce deaths and AIDS-related events, especially among those with profound immunosuppression?	To improve programme performance and outcomes; to collect data to guide policy recommendations on specific interventions	Observational qualitative	TB, HIV, mother and child, and primary healthcare facilities	Setting specific; across settings with analytical approach needed to assess for heterogeneity	Regional, national, local
What is the feasibility, effectiveness and impact of innovative measures to increase HIV testing, such as home-based HIV rapid tests, among those with presumptive and confirmed TB and their relatives?	To improve programme performance and outcomes	Observational qualitative; interventional quantitative	Home-based care programmes and communities	Setting specific	National, local

**4. Service delivery of collaborative TB/HIV activities**					
What are the best models to integrate and deliver collaborative TB/HIV interventions, including ART, at health sector and community level for people living with HIV, their children and their families?	To improve programme performance and outcomes; to collect data to guide policy recommendations on specific interventions	Observational qualitative; interventional quantitative	TB and HIV healthcare facilities, and communities	Setting specific; across settings with analytical approach needed to assess for heterogeneity	Regional, national, local
What are the best models of community participation (i.e., effective, feasible, acceptable and sustainable) for enhanced TB case finding and early HIV detection to reduce delay in initiation of TB and HIV care, and their impact on reducing TB and HIV transmission?	To improve programme performance and outcomes; to collect data to guide policy recommendations on specific interventions	Observational qualitative; interventional quantitative	Communities affected by both TB and HIV	Setting specific; across settings with analytical approach needed to assess for heterogeneity	Regional, national, local
What are the best models of delivery of collaborative TB/ HIV activities to most-at-rísk populations in settings with different TB and HIV epidemic states?	To improve programme performance and outcomes; to collect data to guide policy recommendations on specific interventions	Observational qualitative; interventional quantitative	TB and HIV healthcare facilities, and communities	Setting specific; across settings with analytical approach needed to assess for heterogeneity	Regional, national, local
How do we determine cost effectiveness of collaborative TB/HIV activities delivered through a community approach and through healthcare facilities?	To improve programme performance and outcomes	Interventional quantitative	TB and HIV healthcare facilities, and communities	Setting specific	National, local
What are the best models of care for drug-resistant TB in HIV prevalent settings (e.g., hospital vs. community-based care) in light of basic public health and individual patient rights?	To improve programme performance and outcomes; to collect data to guide policy recommendations on specific interventions	Observational qualitative; interventional quantitative	TB and HIV healthcare facilities and communities	Across settings with analytical approach needed to assess for heterogeneity; multi settings/countries using generic operational research protocol	Global, regional, national
What are the best models (decentralization of services, mobile systems, etc.) to bring diagnostic services closer to the community and to integrate them into the general health system?	To improve programme performance and outcomes	Observational qualitative and quantitative	TB and HIV care facilities and communities	Setting specific	National, local

**5. Reducing mortality among TB patients living with HIV**					
What are the risk factors associated with death and the causes of death in people living with HIV being treated for TB?	To improve programme performance and outcomes; to collect data to guide policy recommendations on specific interventions	Interventional quantitative	TB and HIV healthcare facilities	Across settings with analytical approach needed to assess for heterogeneity; multi settings/countries using generic operational research protocol	Regional, national, local

**6. Childhood TB and HIV**					
What is the feasibility, effectiveness and impact of current recommendations that are drawn from data from adults and uninfected children to prevent, diagnose and treat TB in children living with HIV?	To improve programme performance and outcomes; to collect data to guide policy recommendations on specific interventions	Interventional quantitative	TB and AIDS programmes	Setting specific and across settings with analytical approach needed to assess for heterogeneity	Global, regional, national
What is the feasibility, effectiveness and impact of Xpert MTB/RIF on sputum samples and other specimens to diagnose TB among children living with HIV?	To improve programme performance and outcomes; to collect data to guide policy recommendations on	Interventional quantitative	TB, HIV and mother and child healthcare facilities up to decentralize level	Multi settings/ countries using generic operational research protocol	Global, regional, national

**7. Other critical areas**					
***7.1 Monitoring and evaluation***					
What are the best strategies to cross check, reconcile and validate data from TB and HIV/AIDS programme to accurately document the implementation of TB/HIV collaborative activities?	To improve programme performance and outcomes	Observational qualitative and quantitative	TB and AIDS programmes	Setting specific	National, local
***7.2 Access to care***					
What are the barriers to care for people living with HIV, their children and families from a patient and healthcare worker's perspective, and how can these barriers be addressed?	To improve programme performance and outcomes	Observational qualitative	TB and HIV care facilities and communities	Setting specific	National, local
***7.3 Civil society and public-private sector engagement***					
What is the best way to engage communities and civil society in research for better research outcomes?	To improve programme performance and outcomes; to collect data to guide policy recommendations on specific interventions	Observational qualitative	TB and HIV civil society and communities	Setting specific; multi settings/countries using generic operational research protocol	Global, regional, national
***7.4 Capacity building for operational research***					
How can the engagement of HIV healthcare providers contribute to TB control and improve access to care for vulnerable populations, and what is their capacity to introduce new tools for TB control?	To improve programme performance and outcomes	Observational qualitative and quantitative	HIV public and private healthcare providers	Setting specific	National, local
How do we enhance capacity building for operational research within programmes in countries with high burdens of TB and HIV diseases, and what is the impact of operational research training modules?	To improve programme performance and outcomes; to collect data to guide policy recommendations on specific interventions	Observational qualitative and interventional quantitative	TB and AIDS programmes	Setting specific and across settings with analytical approach needed to assess for heterogeneity	Global, regional, national

### Prevention of TB among people living with HIV

Meta-analyses of randomized controlled trials have shown that IPT reduces the risk of active TB by 33% in people living with HIV, irrespective of skin test result, and by up to 64% in those with a positive tuberculin skin test [20,21]. Despite the substantial amount of clinical research that showed the benefits of IPT among people living with HIV, several barriers and operational challenges have hampered its roll out [22]. One of these is the difficulty of reliably excluding active TB before preventive therapy, with subsequent concern about the possibility of selecting for isoniazid resistant *M. tuberculosis* strains and eventually multi-drug-resistant strains [23]. WHO has recently developed simplified TB screening algorithms for adults and children living with HIV that will promote the delivery of IPT for people living with HIV[24].

Although the adult algorithm was based on an individual participant data meta-analysis of more than 9000 patients [25], the children algorithm mostly relied on expert opinion, due to lack of evidence. Concomitant to intensifying the search for a simple and rapid TB diagnostic tool, the implementation of these algorithms need to be scaled up and their feasibility, effectiveness and impact investigated by national AIDS and TB programmes.

Clinicians are faced with challenges on how to maintain adherence to IPT: studies from Malawi, South Africa and Zambia have shown that adherence varies from 24% to 59% [26]. Concerns also exist on the need and feasibility of monitoring IPT in terms of toxicity and occurrence of TB disease [27]. Recent data from more than 24,000 South African patients showed, however, very low rates of IPT-related adverse events (particularly clinical hepato-toxicity: 0.07%), and suggested that monitoring based on clinical symptoms was sufficient [28]. These encouraging results need to be confirmed in other populations.

Another important question is the duration of preventive therapy. Results from two recent clinical trials from Botswana [29] and South Africa [30] suggest an increased benefit of IPT when given for 36 months or longer, compared with six months, particularly in people who are tuberculin skin test positive. However, a clinical trial reported no difference between 36 and six months of extended use of isoniazid and ethambutol in India [31]. The feasibility of life-long IPT under routine conditions and the factors related to it, as well as the timing of initiation in relation to ART, which also greatly reduces TB incidence [32], need to be further investigated. From a programme perspective, it is important to determine which programmes or services (HIV, TB, mother and child health, or others) will best coordinate nationwide implementation of IPT and ensure that it is included in the comprehensive package of care for people living with HIV [24].

### Implementation of infection control measures

In addition to preventive chemotherapy, efficient infection control measures at facility level are crucial to prevent the transmission of *M. tuberculosis* among people living with HIV [33]*. The*se include: administrative control measures, such as triage; separation of infectious cases; control of pathogens’ spread and reduced hospital stay; environmental control with natural or mechanical ventilation systems and the use of ultraviolet germicidal irradiation; and personal protective protection for healthcare workers. The identification of the best mix of infection control measures to be implemented and of the best means to monitor these in healthcare facilities (including suitable indicators and methods for detecting the concentration of viable *M. tuberculosis* droplet nuclei in room air) is needed. It will assist TB and AIDS programmes in improving their performance and evaluating their contribution to reducing TB transmission.

### Early diagnosis and treatment of TB among people living with HIV

Delay in TB diagnosis may in part explain the high mortality rates among people living with HIV [34]. The effectiveness, impact and modalities of scaling up existing tools and strategies, such as specimen culturing and clinical algorithms [35], need to be assessed. Similarly, the newly developed, fully automated, cartridge-based nucleic amplification assay, Xpert MTB/RIF, which can detect drug susceptible and rifampicin resistant TB in less than two hours, needs to be rapidly introduced and adapted on a large scale in health services. The sensitivity of a single Xpert MTB/RIF test was 98.2% and 72.5% in smear-positive and smear-negative disease, respectively, and 99.1% in rifampicin resistant TB, and was not affected by the patient’s HIV status [36]. WHO has now recommended Xpert MTB/RIF as the initial diagnostic test for all people living with HIV, as well as for those suspected of having multi-drug-resistant TB [37].

**Figure 1:**
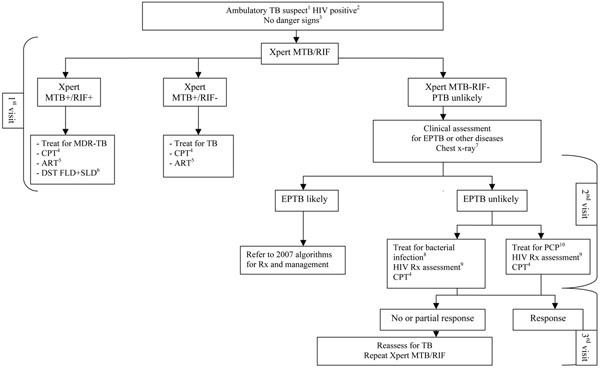
**Algorithm for the diagnosis of tuberculosis in ambulatory HIV-positive patients using Xpert MTB/RIF.**^1^Among adults and adolescents living with HIV, a TB suspect is defined as a person who reports any one of current cough, fever, weight loss or night sweats. Among children living with HIV, a TB suspect is defined as a person who reports one of poor weight gain, fever, current cough, or history of contact with a TB case. ^2^In all persons with unknown HIV status, HIV testing should be performed according to national guidelines. In patients who are HIV negative or remain HIV unknown (e.g., refusal), a TB suspect is defined according to national case definitions. A person with unknown HIV status can still be classified as HIV-positive if there is strong clinical evidence of HIV infection. ^3^The danger signs include any one of: respiratory rate >30/min, temperature >39°C, heart rate >120/min and unable to walk unaided. ^4^CPT = cotrimoxazole preventive therapy. ^5^ART = antiretroviral therapy. All TB patients living with HIV are eligible for ART irrespective of CD4 count. Start TB treatment first, followed by ART as soon as possible within the first 8 weeks of TB treatment. See ART guidelines. ^6^In low MDR-TB prevalence settings, a confirmatory test for rifampicin resistance should be performed. See MDR-TB Xpert MTB/RIF algorithm. ^7^A chest x-ray can assist with the diagnosis of extra-pulmonary TB (e.g., pleural, pericardial) and help assess for other etiologies of respiratory illness. It should only be performed in those settings where the quality of the film and its interpretation are assured. ^8^Antibiotics (except fluoroquinolones) to cover both typical and atypical bacteria should be considered. ^9^An HIV treatment assessment includes WHO clinical staging and/or CD4 count to assess eligibility for antiretroviral therapy. See ART guidelines. ^10^PCP= *Pneumocystis jirovecii* pneumonia.

**Figure 2:**
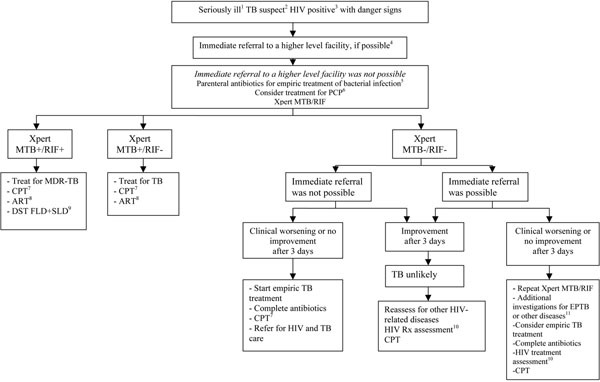
**Algorithm for the diagnosis of tuberculosis in seriously ill HIV-positive patients using Xpert.**^1^Seriously ill refers to the presence of danger signs, including: respiratory rate >30/min, temperature >39°C, heart rate >120/min and unable to walk unaided. ^2^Among adults and adolescents living with HIV, a TB suspect is defined as a person who reports any one of current cough, fever, weight loss or night sweats. Among children living with HIV, a TB suspect is defined as a person who reports one of poor weight gain, fever, current cough, or history of contact with a TB case. ^3^In all persons with unknown HIV status, HIV testing should be performed according to national guidelines. In high HIV prevalent settings, seriously ill patients should be tested using Xpert MTB/RIF as the primary diagnostic test regardless of HIV status. ^4^The highest priority should be to provide the patient with life-sustaining supportive therapy, such as oxygen and parenteral antibiotics. If life-sustaining therapy is not available at the initial point of care, the patient should be transferred immediately to a higher level facility before further diagnostic testing. ^5^Antibiotics (except fluoroquinolones) to cover both typical and atypical bacteria should be considered. ^6^PCP= *Pneumocystis jirovecii* pneumonia. ^7^CPT = cotrimoxazole preventive therapy. ^8^ART = antiretroviral therapy. All TB patients living with HIV are eligible for ART irrespective of CD4 count. Start TB treatment first, followed by ART as soon as possible within the first 8 weeks of TB treatment. See ART guidelines. ^9^In low MDR-TB prevalence setting, a confirmatory test for Rifampicin resistance should be performed. See MDR-TB algorithm. ^10^An HIV treatment assessment includes WHO clinical staging and/or CD4 count to assess eligibility for antiretroviral therapy. See ART guidelines. ^11^Additional investigations for TB may include chest x-ray, liquid culture of sputum, lymph node aspiration for acid-fast bacilli microscopy and culture, abdominal ultrasound. Non-tuberculosis mycobacterial infection should be considered in the differential diagnosis of patients who have a negative Xpert but a sputum or extra-pulmonary specimen with acid-fast bacilli.

The algorithms for the diagnosis of HIV-associated TB, both for ambulatory and seriously ill patients, were revised to include Xpert MTB/RIF and to assist in its implementation in HIV prevalent settings (Figures 1 and 2) [38]. Operational research is crucially needed to assess the feasibility and validity of these revised algorithms. There is an urgent need for collecting further evidence for scaling up Xpert MTB/RIF under programmatic conditions at the various levels of health services, including HIV care facilities, under different epidemiological and resource conditions. Real-time cost-effectiveness data are also needed to inform policy.

The use of ART has been associated with up to 95% reduction in mortality risk among TB patients living with HIV [39], including those with drug-resistant TB [40,41]. Combined ART and TB treatment is more protective than delaying ART after completion of TB treatment [42]. The CAMELIA randomized control trial conducted in Cambodia among 661 HIV-infected TB patients with a median CD4 count of 25 cells/mm^3^ found a 34% reduction in mortality associated with the early initiation of ART two weeks after onset of TB treatment, compared with eight weeks after onset [43]. Similarly the STRIDE and SAPIT trials showed that combined and earlier ART and TB treatment (two to four weeks after TB treatment initiation) reduced deaths and AIDS-related events by 42% and 68%, respectively, among those with CD4 counts of less than 50 cells/mm^3^ when compared with ART initiation within eight to 12 weeks of TB treatment [44,45]. These results confirm current WHO recommendations to start ART as soon as possible in the course of TB treatment [46], especially in people with profound immunosuppression. Operational research to inform national AIDS and TB programmes on how best to implement these recommendations and scale up the uptake of ART in TB patients living with HIV is urgently needed and should include the assessment of various service delivery models for collaborative TB/HIV activities.

A prerequisite for improved early diagnosis and treatment of TB among people living with HIV is knowledge of HIV status. HIV testing for those with confirmed and presumptive TB and their contacts yields a high number of new diagnoses of HIV [47-50], and should be encouraged. Any innovative measure to increase the uptake of HIV testing, such as the home-based rapid test [51], needs to be assessed and standardized for TB patients, TB suspects and their relatives.

### Service delivery of collaborative TB/HIV activities

Integration of TB and HIV services to deliver collaborative TB/HIV activities is important in settings where most TB patients also have HIV infection and therefore are in need of ART. A recent systematic review has shown that different models of integration of services are being implemented: (i) TB services refer patients for HIV testing and treatment; (ii) HIV services refer people living with HIV for tuberculosis screening and treatment; (iii) TB services test patients for HIV and refer them for treatment; (iv) HIV services screen for tuberculosis but refer patients for treatment; and (v) TB and HIV services are provided at a single facility [52]. However, the best delivery model is unknown and it is unlikely that a “one-size-fits-all” approach will work.

Ways to decentralize TB and HIV services into primary healthcare, as well as mother and child healthcare facilities, should also be investigated. Operational research is needed to provide evidence on best service delivery models and on their effectiveness, so as to enhance the uptake of TB and HIV prevention, diagnosis and treatment for people affected by, and at risk for, both diseases. The impact of the models on patients’ outcomes also needs to be investigated [52]. Specific attention should be paid to evaluating these questions in special populations, such as children, women, prisoners and injecting drug users. Particular emphasis is needed for the integrated delivery of TB and HIV services with harm-reduction services in those settings where drug use is fuelling the HIV epidemic.

Community participation for enhanced TB case finding and early HIV detection to reduce delay in initiation of TB and HIV care also needs to be further investigated by research stakeholders, including national TB and AIDS programmes. TB control in settings with high HIV prevalence will not succeed without reaching out into the community [53], and innovative ways of doing so, such as using mobile vans [54] or health extension workers [55], need to be tested under routine conditions. Identification of the best ways that TB and HIV/AIDS programmes could bring TB prevention, diagnosis and treatment services for both drug-susceptible and drug-resistant TB closer to the community through innovation and decentralization of service is crucial. Finally, cost-effectiveness analyses of the various healthcare- and community-level service delivery models are important to maximize value for money in a time of scarce resources [56].

### Reducing mortality among TB patients living with HIV

In addition to ART, cotrimoxazole preventive therapy (CPT) has been found to reduce mortality in people living with HIV and treated for TB [57]. However, the provision of both ART and CPT has not been fully scaled up, and operational research can assist in determining the best ways to do so. Operational research can also help determine if there are other context-dependant and modifiable risk factors, such as undiagnosed multi-drug-resistant TB, malnutrition and poor treatment adherence, that are associated with death among patients living with HIV on TB treatment despite receiving ART and CPT. Prospective assessment of clinical data could help identify risk factors associated with mortality rates at fixed time points.

### Childhood TB and HIV

Most infants born to HIV-infected mothers still receive the Bacille Calmette-Guérin (BCG) vaccine, as HIV infection is usually not ruled out in the first weeks of life. BCG can cause disseminated mycobacterial disease in HIV-infected children, including those on ART [58], and its efficacy has been questioned in HIV-infected infants up to one year of age [59]. While waiting for a better and safer vaccine to prevent TB, the role of the BCG vaccine in infants living with HIV should be further investigated. Similarly, while isoniazid preventive therapy has been found to reduce the risk of TB disease by 72% and mortality by 56% in children living with HIV [60], little is known about the benefit of IPT in the context of ART, the optimal duration and regimen for preventive TB therapy, the duration of protective effect, and the long-term adverse events among children living with HIV.

In the absence of mycobacterial confirmation, the diagnosis of childhood TB relies on clinical features, exposure history, tuberculin skin test, relevant investigations for suspected pulmonary or extrapulmonary TB, and HIV testing in areas of high HIV prevalence [61]. The use of Xpert MTB/RIF on sputum samples and others specimens to diagnose TB among children living with HIV should be urgently evaluated. Preliminary results show that Xpert MTB/RIF performs better in those children living with HIV than in HIV-negative children (Catharina Boehme, personal communication).

Issues related to combined TB and ART treatment in children with HIV, such as optimal time to initiate ART, drug interactions, overlapping side effects, and diagnosis and management of immune reconstitution inflammatory syndrome, have been poorly investigated. Current recommendations are often drawn from data from HIV-un-infected children and adults [62] or rely on expert opinion. There is an urgent need to conduct operational research to assess the feasibility, effectiveness and impact of these recommendations and further inform national and international guidance on TB treatment, but also prevention and diagnosis in infants and children living with HIV.

Lastly, control of HIV-associated TB in children will not succeed without tackling their mothers. In the prevention of mother to child transmission of HIV, prevention of TB is crucial since maternal TB has been associated with increased vertical transmission of HIV [63].

### Other critical areas

#### Monitoring and evaluation

Functional TB/HIV monitoring and evaluation system with standardized indicators is crucial to inform policy and programming. The capacity to cross check and reconcile accurate data between TB and AIDS programmes will depend on the existence of standardized and integrated recording and reporting formats (patient cards, clinical files and registers), and will assist in identifying recording and reporting gaps so as to improve the monitoring and evaluation of collaborative TB/HIV activities. Effective and regular joint supervision by TB and AIDS programmes may greatly improve the process.

#### Access to care

There are several structural and social barriers of access to TB and HIV services for people living with HIV [64]. Qualitative research to better understand the barriers to TB and HIV care for people living with HIV, their children and their families, both from a patient and health care worker’s perspective, is important in improving access to and use of diagnostic services.

#### Civil society and public-private sector engagement

Enhancing the role of civil society organizations in implementation and research [65] and the promotion of sustainable collaboration between all public and private practitioners for TB care and control are essential for scaling up collaborative TB/HIV activities [66]. The best way to achieve these goals needs to be worked out.

#### Capacity building for operational research

Finally, enhanced capacity building for operational research within programmes in countries with high burdens of TB and HIV diseases and a transfer of north-to-south expertise is urgently needed [67]. Trainings and courses on operational research for collaborative TB/HIV activities need to take this into consideration, and ensure that there is leadership by researchers, programme managers and other stakeholders based in the countries afflicted by the dual epidemic. This will result in ownership of the research that will facilitate its translation into policy and programming.

## Conclusions

Promoting research is a key component of the Stop TB Strategy, which includes conducting “programme-based operational research” and “research on introducing new tools into practice” [68]. The importance of programme-based operational research is increasingly recognized, as exemplified through the insertion of a concise Operational Research section in the new Global Plan to Stop TB 2011-2015 [4], and was recently identified as a major area in which global action is urgently needed [69].

In this paper, we have reviewed the main operational research priorities that need to be addressed to improve the implementation of collaborative TB/HIV programme activities in areas affected by both epidemics [16,17,19]. We have seen how operational research is crucial in answering the translation gaps in implementing combinations of existing and new tools and technologies to improve the prevention, early diagnosis and prompt treatment of TB among people living with HIV. Optimal control of TB in high HIV burden areas requires strong collaborative TB/HIV interventions through sound policy and programme environments that give due consideration to the local context and the respective epidemiology of TB and HIV. Moreover, since both TB and HIV control activities are placed within the larger frame of health services, due consideration should also be given to the health system infrastructure, as it has implications in terms of general health system functions (e.g., finances, regulations, policy development, management of human resources, procurement of drugs and supplies, and maintenance of health infrastructure) [70]. It is therefore important to address these questions within the larger context of health services research to identify measures that would facilitate wider uptake and scaling up of collaborative TB/HIV interventions for prevention, diagnosis and treatment, through effective service delivery models, including community-based interventions [71]

In this respect, national TB and AIDS programmes should develop their operational research agendas and conduct the research they consider crucial for improving TB and HIV control in their settings in collaboration with research stakeholders. Support from academic institutions and international organizations is essential for implementing research questions that aim to assess new tools and strategies and to inform global policy, as well as to build local capacity in conducting operational research. Financial resources to implement these operational research questions should be mobilized and drawn from existing funding mechanisms (among others, the Global Fund to fight AIDS, Tuberculosis and Malaria, the President’s Emergency Plan for AIDS Relief, and the Bill & Melinda Gates Foundation), and new funding mechanisms need to be sought. Enhancing fund allocation for operational research by national governments of countries highly affected by both diseases and ensuring the expedited translation of results of the research into policy and programming are points of equally crucial importance.

## Competing interests

The authors declare no financial interests. The authors are staff members of the World Health Organization. The authors alone are responsible for the views expressed in this publication and they do not necessarily represent the decisions or policies of the World Health Organization.

## Authors’ contributions

DS was involved in drafting the manuscript. HG and CL were involved in revising the manuscript for important intellectual content.
